# Molecular networking prospection and characterization of terpenoids and C_15_-acetogenins in Brazilian seaweed extracts†

**DOI:** 10.1039/c8ra02802h

**Published:** 2018-08-21

**Authors:** Ana Cláudia Philippus, Gabriele A. Zatelli, Tauana Wanke, Maria Gabriela de A. Barros, Satomy A. Kami, Cintia Lhullier, Lorene Armstrong, Louis P. Sandjo, Miriam Falkenberg

**Affiliations:** Federal University of Santa Catarina (UFSC), Postgraduate Program in Pharmacy, Health Sciences Center Florianópolis 88040-900 Brazil miriam.falkenberg@gmail.com

## Abstract

Molecular networking (MN) can efficiently dereplicate extracts and pure compounds. Red algae of the genus *Laurencia* are rich in halogenated secondary metabolites, mainly sesquiterpenes and C_15_-acetogenins. Brown algae of the genus *Dictyopteris* produce mainly C_11_-hydrocarbons, sesquiterpenes and sulfur-containing compounds, while *Dictyota* and *Canistrocarpus* are reported to contain mainly diterpenes. This study performs an exploratory MN analysis of 14 extracts from algae collected in Brazil (including the oceanic islands) and characterizes the secondary metabolites from the analyzed species. The extracts and some isolated metabolites were analyzed by LC-MS using the FastDDA algorithm, and the MS/MS spectra were submitted to GNPS and displayed in Cytoscape 3.5.1. The GNPS platform generated 68 individual nodes and nine family networks. The MN exploratory analysis indicated chemical differences among species, and also in sampling sites for the same species. For some extracts, it was possible to identify mass values that could correspond to terpenoids and C_15_-acetogenins that have already been isolated from those or related species. An interesting chemodiversity was highlighted between *Laurencia catarinensis* from two nearby islands, and this was revealed and was also suggested by the family networks. Many nodes in the MN could not be characterized, and these metabolites can be used as targets for isolation in future works.

## Abstract 2

Molecular networking of Brazilian marine algae.

## Graphical abstract

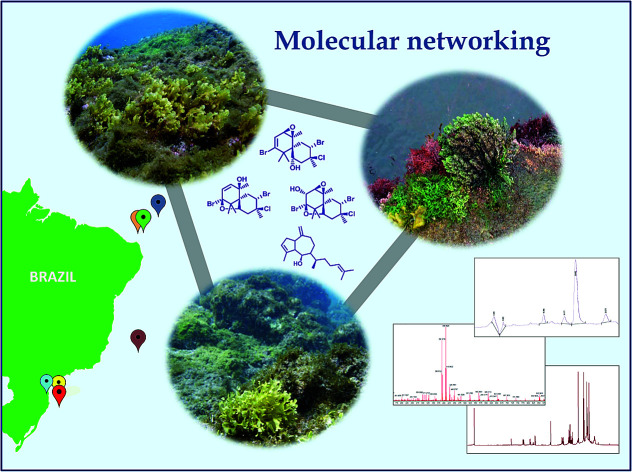

## Introduction

Recently, new approaches have been developed to guide the isolation of new metabolites, with reduced costs and effort. MS/MS-based Global Natural Products Social (GNPS) Molecular Networking (MN) is an example of a platform that can be easily integrated into natural product workflows, to efficiently dereplicate extracts and pure compounds.^1,2^ Using a special computational algorithm to compare the degree of spectral similarity between the MS/MS spectra of different compounds, this technique provides a visual overview of all the ions that were detected and fragmented in the MS experiment.^3^ The key is based on structurally related natural products that share similar tandem mass fragmentation patterns, and molecular families that tend to cluster together and can be visualized as a network.^2,4^ This dereplication strategy has been widely used for the identification of marine and microorganism-derived natural products.^4,5^

The marine environment represents approximately 70% of the earth's surface, and has a huge biodiversity, characterized by a wide chemical diversity of natural products.^6,7^ The Brazilian maritime area, known as the “Blue Amazon”, has one of the longest coastlines in the world, and five oceanic islands.^8^ However, it is still under-explored as a source of natural products. This marine area is largely located in tropical zone, contributing to the development of a rich and diverse marine biota. Many marine algae species of the genera *Laurencia*, *Dictyota*, *Canistrocarpus* and *Dictyopteris*, can be found in this area.

While red algae of the genus *Laurencia* J. V. Lamouroux are rich in halogenated secondary metabolites, belonging mainly to the sesquiterpenes, diterpenes, triterpenes, and C_15_-acetogenins classes,^9^ brown alga of the genus *Dictyopteris* produce mainly C_11_-hydrocarbons, sesquiterpenes and sulfur-containing compounds.^10^ In addition, *Dictyota* and *Canistrocarpus* are chemically composed of diterpenes.^11–16^ The metabolites produced by algae mainly serve as chemical defenses against herbivorous or pathogenic micro-organisms.^16^ Many of these metabolites have been confirmed as presenting anticoagulant, antifouling, antimicrobial, antioxidant, herbivore-deterrent and cytotoxic activities.^9, 10, 17–20^

This study reports an exploratory MN analysis of fourteen algae extracts of algae collected mostly from Brazilian oceanic islands and the characterization of terpenoids and C_15_-acetogenins from some of the analyzed species.

## Results and discussion

**Fig. 1:**
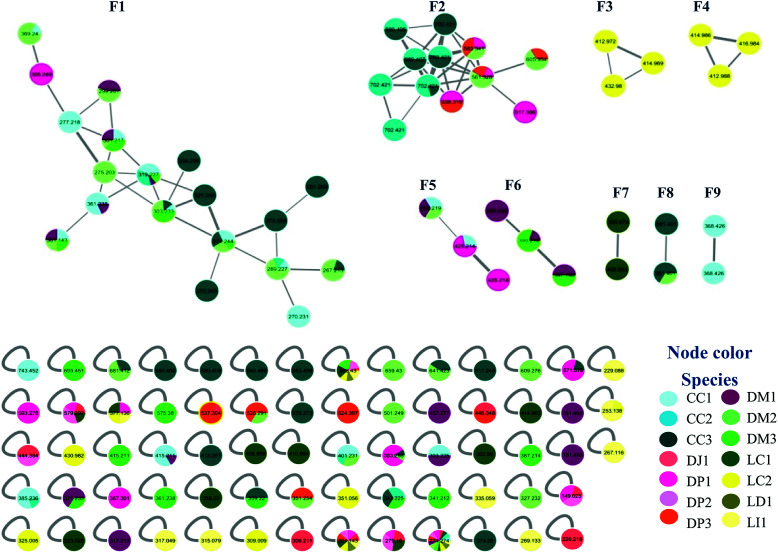
Molecular networking of Brazilian algae extracts.

Samples were collected in 14 different locations. These samples were identified as belonging to seven species (Table 1). Analysis of the MN data revealed that 10 of the collected samples were distributed in nine family networks (F1–F9) of which: F1 presented 19 nodes, F2 showed 12 nodes, F3–F6 contained three nodes, and F7–F9 comprised two nodes (Fig. 1). Metabolites from extracts of red and brown algae clustered in different family networks, as was expected. Moreover, two extracts of brown (DJ1, DP2) and two of red algae (LD3 and LI4) were not included in any family network. The entire network was formed by 117 nodes, including 68 individual nodes.

**Table 1:** Description of the samples collected in Brazilian coast and oceanic islands

Species	Code	Collection site	Date				
*Canistrocarpus cervicornis*	CC1	Rocas Atoll, Rio Grande do Norte/Brazil	03/2015^a^				
	CC2	Rocas Atoll, Rio Grande do Norte/Brazil	03/2015^a^				
	CC3	Rocas Atoll, Rio Grande do Norte/Brazil	03/2015^a^				
*Dictyopteris jolyana*	DJ1	Fernando de Noronha Archipelago, Pernambuco/Brazil	10/2014				
*Dictyopteris plagiogramma*	DP1	Fernando de Noronha Archipelago, Pernambuco/Brazil	11/2014				
	DP2	Fernando de Noronha Archipelago, Pernambuco/Brazil	11/2014				
	DP3	Rocas Atoll, Rio Grande do Norte/Brazil	05/2016				
*Dicyota mertensii*	DM1	Trindade Island, Espírito Santo/Brazil	08/2014				
	DM2	Fernando de Noronha Archipelago, Pernambuco/Brazil	10/2016				
	DM3	São Pedro and São Paulo Archipelago, Pernambuco/Brazil	10/2016				
*Laurencia catarinensis*	LC1	Xavier Island, Santa Catarina/Brazil	06/2015				
	LC2	Arvoredo Island Santa Catarina/Brazil	02/2008				
*Laurencia dendroidea*	LD3	Bombinhas, Santa Catarina/Brazil	11/2015				
*Laurencia intricata*	LI4	Rocas Atoll Rio Grande do Norte/Brazil	03/2016				

a Collection performed in different points.

**Fig. 2:**
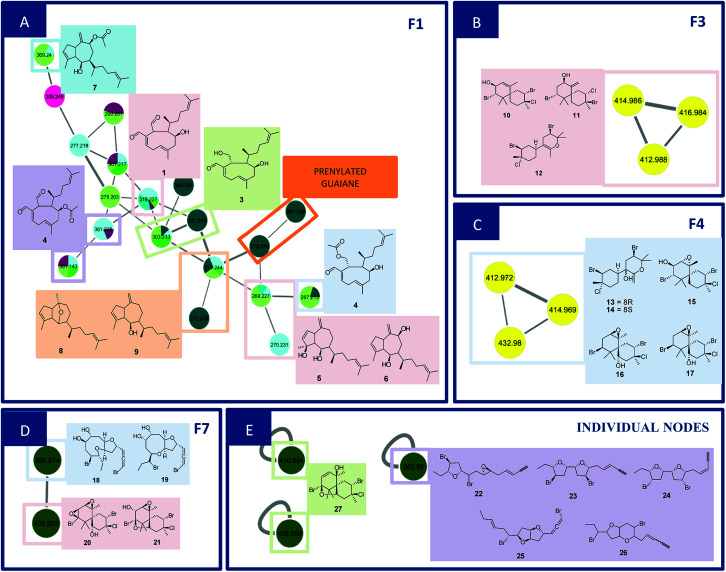
Family 1 (A), 3 (B), 4 (C), 7 (D) and individual nodes (E) and related compounds.

F1 (Fig. 2A) showed a node with *m*/*z* 319.2274 [C_20_H_30_O_3_ + H]^+^ (calcd 319.2273) corresponding to the peak at 8.96 min in the LC-MS data for the CC1, CC2, DM1 and DM3 extracts. Fragments were also observed resulting from loss of H_2_O (18 Da), and two H_2_O molecules (36 Da) at *m*/*z* 301.2172 [C_20_H_28_O_2_ + H]^+^ (calcd 301.2167) and *m*/*z* 283.2065 [C_20_H_26_O_2_ + H]^+^ (calcd 283.2062). Its MS/MS data matches those of the xeniane diterpene 4β-hydroxydictyodial (1).^15^ Similar MS^2^ behavior was observed for the node at *m*/*z* 361.2383 [C_22_H_32_O_4_ + H]^+^ (calcd 361.2379) detected at 10.48 min in the CC1 and DM1 LC-MS data. This metabolite differs from 1 by 42 Da, suggesting that it is an acetylated derivative. The above data, together with the data reported in the literature, suggest the structure of 4β-acetoxydictyodial A (2), another xeniane diterpene.^14^

The third node *m*/*z* 321.2441 [C_20_H_32_O_3_ + H]^+^ (calcd 321.2429) (RT 7.74 min) lost H_2_O (18 Da) to afford *m*/*z* 303.2332 [C_20_H_30_O_2_ + H]^+^ (calcd 303.2324). This precursor was found in the CC1, CC3, DM2 and DM3 LC-MS data. Moreover, daughter ions were observed at *m*/*z* 285.2236 [C_20_H_28_O + H]^+^ (calcd 285.2218) and *m*/*z* 267.2100 [C_20_H_26_ + H]^+^ (calcd 267.2113). Both ions were produced from the loss of 2 (36 Da) and 3 (54 Da) H_2_O molecules, respectively. These data suggest another xeniane diterpene 18,4-dihydroxydictyo-19-al A (3).^15^

The node *m*/*z* 267.2134, detected at 7.32 min in the CC3 and DM1 LC-MS spectra, turned out to be a base peak ion, which was the fragment of the precursor *m*/*z* 385.2359 [C_22_H_34_O_4_ + Na]^+^ (calcd 385.2355) as observed in the node mass spectrum. Fragments resulting from the loss of NaOH (40 Da), [NaOH (40 Da) and H_2_O (18 Da)], [NaOH (40 Da), H_2_O (18 Da) and ketene (42 Da)], and [NaOH (40 Da), 2H_2_O (36 Da) and ketene (42 Da)], were obtained at *m*/*z* 345.2441 [C_22_H_33_O_3_]^+^ (calcd 345.2424), *m*/*z* 327.2324 [C_22_H_31_O_2_]^+^ (cald 327.2319), *m*/*z* 285.2236 [C_22_H_29_O]^+^ (calcd 285.2213) and *m*/*z* 267.2134 [C_22_H_27_]^+^ (calcd 267.2107), respectively. The literature data suggested another xeniane congener, and the structure of 18-acetoxy-4-hydroxydictyo-19-al A (4) was considered a possibility.^15^

Another F1 node at *m*/*z* 269.2265 (detected at 11.84 min in CC1, CC2, and DM2) is also a base peak ion of the precursor at *m*/*z* 327.2287 [C_20_H_32_O_2_ + Na]^+^ (calcd 327.2300). This precursor produced *m*/*z* 287.2375 [C_20_H_31_O]^+^ (calcd 287.2369) and *m*/*z* 269.2265 [C_20_H_28_ + H]^+^ (calcd 269.2264) after losing NaOH (40 Da), [NaOH (40 Da) and H_2_O (18 Da)]. The information outlined above, in conjunction with the literature search led to two previously reported compounds. Since the completed structure is not always possible using tandem mass data alone, dictyotadiol (5) and dictyol B (6) were suggested as potential structures.^14,16^ Similar MS/MS behavior was observed for the node at *m*/*z* 369.2395 [C_22_H_34_O_3_ + Na]^+^ (calcd 369.2406) that corresponded to the peak at 12.00 min in the CC1 and DM2 LC-MS data. This metabolite differs from 5 and 6 by 42 Da suggesting that it is an acetylated derivative. The above data, together with the data reported in the literature led to the structure of dictyol B acetate (7), another guaiane prenylated diterpene.^16^

The node at *m*/*z* 271.2439 [C_20_H_31_O + H]^+^ (13.09 min in CC1, CC2, DM2 and DM3) represents a base peak ion formed when the precursor at *m*/*z* 289.2550 [C_20_H_32_O + H]^+^ (calcd 289.2531) lost H_2_O (18 Da). The literature search, together with the above information, led to the hypotheses of two prenylated guaiane diterpenes, namely, dictyoxide (8) and pachydictyol A (9).^13^ Although two metabolites already reported in the literature were suggested, the node could refer to 9, since this compound was later isolated from the extract DM2.

The node *m*/*z* 291.2691 [C_20_H_34_O + H]^+^ (RT 4.15 min in CC3) afforded *m*/*z* 273.2586 [C_20_H_32_ + H]^+^ (calcd 273.2582) as a fragment ion after losing H_2_O (18 Da). The literature search resulted in a no-hit compound as chemotaxonomy was considered. However, the mass spectrum of this node gave the precursor ion with 2 Da lighter than 8 and 9 suggesting a prenylated guaiane diterpene analog.

In general, MN clustered in F1 metabolites are mainly from the brown algae *Canistrocarpus cervicornis* and *Dictyota mertensii* (Fig. 1), with similar fragmentation patterns to the diterpenes already reported for the *Dictyota* species. Although the literature for *C. cervicornis* regards the presence of dolastane and secodolastane, curiously only the guaiane prenylated and xeniane types were observed in the nodes of F1. This suggest that *C. cervicornis* from Rocas Atoll is chemically diverse from other specimens collected on the Brazilian Coast. Compounds 8 and 9 were previously identified in *D. mertensii*,^12,13^ while 1–4 and 6 were obtained from *D. crenulata*^14,15^ and 5 and 7 were identified in *D. dichotoma*.^21^

The nodes with *m*/*z* 270.2309, *m*/*z* 272.2452 and *m*/*z* 304.2349 were found in LC-MS, but these peaks correspond to isotopic patterns of the nodes with *m*/*z* 269.2265, *m*/*z* 271.2439 and *m*/*z* 303.2332, respectively.

F2 was characterized by heavier mass values for the nodes, however none of the structures was established.

Families F3 (Fig. 2B) and F4 (Fig. 2C) are formed by three nodes each and composed only of metabolites produced by *L. catarinensis* from Arvoredo Island (LC2). F3 contains the nodes *m*/*z* 412.9883, *m*/*z* 414.9859 and *m*/*z* 416.9840. These three mass values were found in the same spectrum, and belong to the same metabolite. This mass spectrum showed *m*/*z* 412.9883, 414.9859, 416.9840 and 418.9828 at a ratio of 3 : 7 : 5 : 1, corresponding to the isotopic pattern of a metabolite containing 2 bromine atoms and one chlorine atom. Two metabolites were observed at 7.74 and 8.11 min with this particular isotopic pattern and the mass value of *m*/*z* 412.9883. The elemental composition [C_15_H_23_Br_2_ClO + H]^+^ (calcd *m*/*z* 412.9882) is compatible with halogenated marine sesquiterpenes. Its fragmentation spectrum revealed ions at *m*/*z* 333.0627/335.0609/337.0566 (4 : 5 : 1), *m*/*z* 253.1281/255.0815 (3 : 1), *m*/*z* 217.1458; the first fragment was formed after the loss of HBr (80 Da), while the second fragment resulted from the elimination of 2 HBr (160 Da); the last fragment was produced after the elimination of 2 HBr and HCl (196 Da). The foregoing data in conjunction to the data reported in the literature led to the structure of some chamigrane-type sesquiterpenes, such as 9-hydroxy-4,10-dibromo-3-chloro-α-chamigrene (10)^22–24^ and 2,10-dibromo-3-chloro-8-hydroxy-β-chamigrene (11),^25^ along to the irregular rearranged bisabolane-related sesquiterpene laucapyranoid A (12).^26^ The metabolite detected at 7.74 and 8.11 min might be related, due to similar fragmentation behavior. Compound 10 was reported for many *Laurencia* species, such as *L. nidifica*,^22^*L. nipponica*^23^ and *L. pacifica*,^24^ while compound 11 was reported only for *L. nipponica*.^25^

F4 presented similar feature to F3, showing three nodes at *m*/*z* 412.9717, *m*/*z* 414.9692 and *m*/*z* 432.9804. These three mass values were found in the same spectrum and belong to the same metabolite. They originated from the precursor at *m*/*z* 428.9848, which appeared alongside *m*/*z* 430.9824, 432.9804 and 434.9787 at a ratio of 3 : 7 : 5 : 1, corresponding to the isotopic pattern of a metabolite containing 2 bromine atoms and one chlorine atom. This precursor ion lost H_2_O (18 Da) and produced the fragment at *m*/*z* 410.9749, with the same isotopic pattern. Three isomeric metabolites were found with this particular isotopic pattern and the mass value of *m*/*z* 428.9831 at 7.04, 7.45 and 9.99 min. Their elemental composition [C_15_H_23_Br_2_ClO_2_ + H]^+^ (calcd *m*/*z* 428.9831) supported the presence of halogens in these marine sesquiterpenes. A literature search led to the structure of sesquiterpenes related to laucapyranoids B (13), C (14),^26^ 4,10-dibromo-3-chloro-7,8-epoxy-9-hydroxychamigrane (15),^26^ 4,10-dibromo-3-chloro-7,8-epoxychamigrane (16)^27^ and 4,10-dibromo-3-chloro-7,8-epoxy-5-hydroxychamigrane (17).^28^ Compounds 13 and 14 were already reported for *L. caespitosa*,^26^ while compound 15 was found in *L. glomerata*^28^ and *L. flagellifera*,^29^ and 17 in *L. scoparia*.^28^

None of the structure was established for F5 and F6.

F7 (Fig. 2D) contained only two nodes at *m*/*z* 390.9736 and *m*/*z* 408.9829. The first ion was generated from the precursor *m*/*z* 424.9951 (RT 5.46 min in LC-MS of LC1), which appeared alongside *m*/*z* 426.9961 and 428.9933 at a ratio of 1 : 2 : 1. This isotopic proportion indicated the presence of 2 bromine atoms in this compound. Its elemental composition [C_15_H_22_Br_2_O_4_ + H]^+^ (calcd *m*/*z* 424.9963) supported the brominated nature of this metabolite. The MS spectrum revealed ions at *m*/*z* 406.9875/408.9829/410.9832 (1 : 2 : 1) and *m*/*z* 388.9743/390.9736/392.9700 (1 : 2 : 1), furnished respectively by a successive loss of H_2_O (18 Da), and 2H_2_O (36 Da). Moreover, further fragment ions were observed in the MS^2^ spectrum at *m*/*z* 327.0549 [M + H–H_2_O–Br]^+^, *m*/*z* 309.0506 [M + H–2H_2_O–Br]^+^, *m*/*z* 271.0301 [M + H–2H_2_O–(1-bromopropa-1,2-diene)]^+^ and *m*/*z* 240.0135 [M + H–2H_2_O–(1-bromopropa-1,2-diene)–C_2_H_6_]^+^. The foregoing data in conjunction to those reported in the literature led to the structural hypotheses of two brominated C_15_-acetogenins namely laurendecumallenes A (18) and B (19), which has already been reported in the chemical study of *L. decumbens*.^30^

Node *m*/*z* 408.9829, in turn, was a fragment ion of the precursor *m*/*z* 442.9620 (RT 7.93 min), which appeared in the mass spectrum alongside *m*/*z* 444.9616, 446.9571 and 448.9570 at a ratio of 3 : 7 : 5 : 1, corresponding to the isotopic pattern of a metabolite containing 2 bromine atoms and one chlorine atom. Its elemental composition [C_15_H_21_Br_2_ClO_3_+H]^+^ (calcd *m*/*z* 442.9624) supported the halogenated nature of this marine sesquiterpene. The MS spectrum showed a fragment ion at *m*/*z* 406.9875/408.9829/410.9832 (1 : 2 : 1) after loss of HCl (36 Da). Furthermore, the MS^2^ spectrum gave a fragment ion at *m*/*z* 309.0487 after eliminating 2H_2_O (36 Da), HBr (80 Da) and HCl (36 Da). As reported for F3 and F4, these findings indicated a chamigrane sesquiterpene type, which was compatible with the structures of prepacifenol epoxide (20)^39^ and johnstonol (21).^22^ Since 20 and 21 were isolated from LC1 extract and presented the same retention times and fragmentation patterns, it is possible that this node corresponds to both compounds. Nevertheless, according to the literature, prepacifenol epoxide may be converted into johnstonol.^22^ Compound 20 was also isolated from *L. composita*,^31^*L. johnstonii*,^20^*L. nidifica*,^22^*L. okamurai*,^32^ while compound 21 was reported for *L. nidifica*,^22^*L. okamurai*^32^ and *L. pacifica*.^33^

For F8 and F9, it was also not possible to establish any structure.

The individual node with *m*/*z* 392.9902 also corresponded to a metabolite produced by *L. catarinensis* from Xavier Island (LC1 extract). This mass spectrum showed *m*/*z* 390.9898, 392.9902 and 394.9876 at ratio of 1 : 2 : 1, corresponding to the isotopic pattern of a metabolite containing 2 bromine atoms. Two isomeric metabolites were observed at 4.96 and 6.39 min with this particular isotopic pattern and the mass value of *m*/*z* 390.9898. Its elemental composition [C_15_H_20_Br_2_O_2_ + H]^+^ (calcd *m*/*z* 392.9888) confirmed a dibrominated metabolite. The literature search led to the hypotheses of some C_15_-acetogenins containing five-membered cyclic ethers ring, such as laureepoxide (22) (one tetrahydrofuran ring),^34^ (3*E*)-elatenyne (23) and elatenyne (24) (two isolated tetrahydrofuran rings),^30,35^ and kumausallene (25) (two fused tetrahydrofuran rings),^25^ along with laurobtusin (26), an acetogenin-containing six-membered cyclic ether ring.^36^ Compound 22 was reported to *L. nipponica*,^34^23 to *L. majuscula*,^35^24 to *L. decumbens*^30^ and *L. elata*,^37^25 to *L. nipponica*^25^ and 26 to *L. obtusa*.^36^

The individual nodes at *m*/*z* 408.9581 and 410.9542 correspond to the metabolite from LC1 extract with a retention time at 9.80 min. These mass values were found in the same spectrum alongside *m*/*z* 412.9509 and *m*/*z* 414.9525 at a ratio of 3 : 7 : 5 : 1 corresponding to the isotopic pattern of a metabolite containing 2 bromine atoms and one chlorine atom. This ion was produced by the precursor at *m*/*z* as observed in the mass spectrum. Loss of H_2_O [C_15_H_21_Br_2_ClO_2_ − H_2_O + H]^+^ (calcd *m*/*z* 428.9654). These data were also indicative of a chamigrane-type sesquiterpene, and this compound was suggested to be pacifenol (27), since MS data and RT were in agreement with those from pacifenol isolated from LC1 (*L*. *catarinensis* from Xavier Island). This metabolite was reported for some *Laurencia* species, such as *L. filiformis*,^38^*L. majuscula*^39^ and *L. pacifica*.^40^
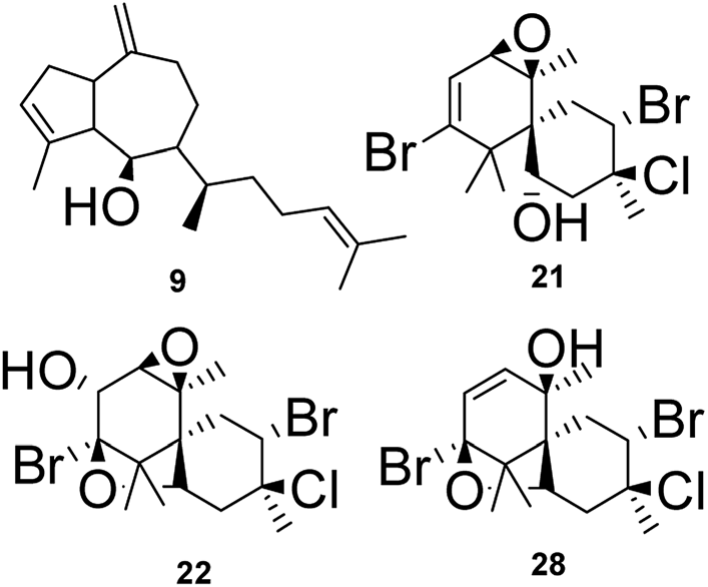


Overall, the diagnosis of the above dereplication data reveals how the marine ecosystem can vary according to geographical location. Marine chemistry is strongly based on the interaction and diversity of animal, plant and microorganism species.^40^ LC1 and LC2, two extracts obtained from the same species of algae collected in relatively close islands from the Southern Brazilian Coast, interestingly supported this fact. For LC1, *L. catarinensis* collected in Xavier Island, three chamigrane-type sesquiterpenes (20, 21 and 27) were characterized; alongside with seven C_15_-acetogenins (18, 19, 22, 23, 24, 25, 26), while for LC2, *L. catarinensis* collected in Arvoredo Island, three compounds related to caespitol (12, 13 and 14) were suggested, together with four chamigrene-type sesquiterpenes (10, 11, 16, 17). Previous chemical work with *L. catarinensis* collected in Arvoredo Island^41^ found mostly caespitol-related metabolites, but no C_15-_acetogenins. Meanwhile, from the ongoing investigation of *L. catarinensis* from Xavier Island, the three chamigranes characterized in the extract LC1 have been isolated and structurally identified and some fractions presented NMR data suggestive of C_15_-acetogenins. It was observed that *Canistrocarpus cervicornis* from Rocas Atoll (CC1-3) clustered mostly with *Dictyota mertensii* from three different oceanic islands (DM1-3), while extracts from *Dictyopteris* spp. presented mostly isolated nodes. Nevertheless, the isolation of further metabolites is needed, in order to allow a better evaluation of those hypotheses. It is also possible that some of the discussed nodes correspond to new metabolites with the same molecular formula structures and similar fragmentation patterns to compounds already reported in the literature.

Previous studies on the *Laurencia* species also showed a different chemical composition in relation to the collection sites.^42,43^ Prevezols A and B, alongside acetogenins were identified from *L. obtusa* collected in Greece,^44,45^ while the same species collected on the Italian coast produced obtusalenes V, VI, VII and IX.^45^ Furthermore, *L. microcladia* from some Greek islands showed different chemical profiles according to the collection sites.^46–49^

No compound could be identified in the extracts of *Dictyopteris* spp., *Laurencia dendroidea* and *L. intricata*.

## Experimental

### Chemicals and materials

Acetonitrile (HPLC grade) and formic acid were purchased from Tedia (São Paulo, Brazil), while ethyl acetate, dichloromethane, hexane and methanol were provided by Vetec Química Fina Ltda (São Paulo, Brazil). Deuterated chloroform was purchased from Cambridge Isotope Laboratories, Inc (Massachusetts, EUA) and silica gel (40–63 μm) from Sigma-Aldrich (Missouri, EUA). Milli-Q system (18.2 MΩ, Milipore, Simplipak. France) was used to produce ultrapure water for aqueous solutions. Filtration of the samples was performed with syringe filter nylon (13 MM; 0.22 μM) from Allcrom (São Paulo, Brazil). Precoated TLC plates (200 mm thickness, silica gel 60 F_254_, Silicycle, Inc.) were used for thin-layer chromatography.

### General experimental procedures

1D and 2D NMR spectra for isolated metabolites were obtained on Bruker DRX 400 MHz spectrometer (Bruker, Massachusetts). HRESIMS data were recorded with Xevo G2-Xs QTof mass spectrometer (Waters Corporation, Milford, MA). Vacuum liquid chromatography (VLC) was performed on silica gel column (40–63 μm; 5 mm × 5.5 mm). Medium pressure liquid chromatography (MPLC) was carried out on Sepacore system (Buchi, Switzerland) using silica gel column (40–63 μm; 15 mm × 920 mm). Solid Phase Extractions (SPE) were performed using Supelclean™ LC-Si SPE cartridges. Semipreparative HPLC was carried out on a Pharmacia LKB 2252 liquid chromatography system equipped with refractive index detector Shodex RI-102, using a Supelcosil column (10 mm × 250 mm × 5 μm, silica).

### Organism collection and extraction

Ten brown (*Dictyopteris plagiogramma*, *Dictyopteris jolyana*, *Dictyota mertensii* and *Canistrocarpus cervicornis*) and four red algae (*Laurencia catarinensis*, *L. dendroidea*, *L. intricata*) samples were obtained from different sites and in different seasons (Table 1), and dried under cold air. The dried material was exhaustively extracted with dichloromethane and methanol (2 : 1) at room temperature and the crude extracts were dried under vacuum.

### LC-MS analysis

The fourteen crude extracts and the isolated metabolites were dissolved in acetonitrile with a final concentration at 2.0 mg mL^−1^, and then filtered over nylon filters. The samples and the blank were injected in an aliquot of 2.0 μL to an Acquity UPLC system equipped with a BEH C18 column (2.1 mm × 50 mm; 1.7 μm), PDA detector and a quaternary bomb. Temperatures of the sample tray and column were set at 20 °C e 40 °C. The flow rate was 0.3 mL min^−1^ of a gradient mobile phase of CH_3_CN/H_2_O (0.1% formic acid): from 80% to 70% of H_2_O in 1 min and then decreasing until 15% of CH_3_CN in 11 min. This proportion lasted 2 min; from 15% to 80% in 1 min; 5 min of 80% of H_2_O were used to return to the initial condition.

The mass spectrometer was set to observe *m*/*z* in a range of 100–1500 in positive ESI mode using the FastDDA algorithm to acquire MS/MS spectra. The capillary voltage was set at 4.0 kV and the cone voltage at 40 V. The cone gas flow was 200 L h^−1^ with an ion source temperature of 80 °C. The desolvation temperature was 300 °C, and the desolvation gas flow was 900 L h^−1^. The analyses were accurate, and leucine enkephalin was used as reference (lock mass *m*/*z* 556.2771). The collision energy was between 25 and 35 eV and argon was used as collision gas for the tandem mass. The software MassLynx V4.1 (Waters Corporation, Milford, USA) was used to process the LC-MS data.

After preliminary MN analysis of the extracts, the same LC-MS method was applied to isolated metabolites for comparison of retention times and MS/MS data.

### Molecular networking

All chromatograms and MS/MS spectra obtained for the 14 crude extracts were digitally converted into .mzML files using MSConvert software (http://www.proteowizard.sourceforge.net), and then submitted to the Global Natural Product Social MN (GNPS) platform (http://gnps.ucsd.edu). The MN was generated by interconnecting MS/MS spectra with precursor ion mass tolerance of 0.002 Da, fragment ion mass tolerance of 0.02 Da, minimum peak intensity equal to 50, a minimum matched of fragment ions and library search minimum matched peaks equal to four. The remaining parameters were kept the same as suggested by the GNPS platform. The MN generated was displayed on Cytoscape 3.6.0.

### Isolation of compounds

The extracts DM2 and LC2 were subjected to vacuum column chromatography on silica gel, using hexane with increasing amounts of EtOAc, followed by EtOAc with increasing amounts of MeOH as the mobile phase, to yield 16 fractions (DA1–DA16) and 20 fractions (LA1–LA20), respectively. Fraction DA2 (10% EtOAc in hexane, 48.9 mg) was fractionated by normal-phase classic column, using hexane with increasing amounts of EtOAc as eluents, to yield 6 fractions (DB1–DB6). Fraction DB3 (17.5 mg) was purified by normal-phase HPLC, using cyclohexane/EtOAc (8 : 2) as eluents, to yield 9 (DC3, 2.2 mg). Fractions LA3 and LA4 (5% and 10% EtOAc in hexane, 345.0 mg) were fractionated by normal-phase MPLC, using hexane with increasing amounts of EtOAc as eluent, to yield 48 fractions (LB1–LB48). Fractions LB20 and LB26 (155.0 mg) were purified by normal-phase MPLC, using hexane with increasing amounts of EtOAc as eluent, to yield 27 (C44–45, 14.2 mg) and 21 (C48–50, 17.4 mg). Fraction LA5 (15% EtOAc in hexane, 114.0 mg) was fractionated by normal-phase MPLC using hexane with increasing amounts of EtOAc as the mobile phase, to yield 75 fractions (LD1–LD75). Fractions LD15, LD16 and D17 (79.0 mg) were purified by normal-phase HPLC, using cyclohexane/EtOAc (95 : 5) as eluents, to yield 20 (LE2, 36.0 mg).

### Data of isolated metabolites

#### Pachydictyol A (9)

White powder; *R*_f_ 0.29, silica gel 60 F_254_, Hex/AcOEt (98.5 : 1.5); ^1^H NMR (CDCl_3_, 300 MHz) and ^13^C NMR (CDCl_3_, 75 MHz) data were in agreement to the literature.^13^

#### Prepacifenol epoxide (20)

White powder; *R*_f_ 0.47, silica gel 60 F_254_, Hex/AcOEt (9 : 1); ^1^H NMR (CDCl_3_, 400 MHz) and ^13^C NMR (CDCl_3_, 75 MHz) data were in agreement to the literature.^39^ Positive ESIMS [M + H]^+^*m*/*z* 442.9628, 444.9620, 446.9571, 448.9523 (calc. for C_15_H_22_Br_2_ClO_3_, 442.9624). RT 7.93 min.

#### Johnstonol (21)

Yellow powder; *R*_f_ 0.25, silica gel 60 F_254_, Hex/AcOEt (9 : 1); ^1^H NMR (CDCl_3_, 400 MHz) and ^13^C NMR (CDCl_3_, 75 MHz) data were in agreement to the literature.^50^ Positive ESIMS [M + H]^+^*m*/*z* 442.9620, 444.9616, 446.9571, 448.9570 (calc. for C_15_H_22_Br_2_ClO_3_, 442.9624). RT 7.93 min.

#### Pacifenol (27)

White powder; *R*_f_ 0.4, silica gel 60 F_254_, Hex/AcOEt (9 : 1); ^1^H NMR (CDCl_3_, 400 MHz) and ^13^C NMR (CDCl_3_, 75 MHz) data were in agreement to the literature.^51^ Positive ESIMS [M − HCl + H]^+^*m*/*z* 408.9576, 410.9532, 412.9537, 414.9507 (calc. for C_15_H_19_Br_2_ClO, 408.9569). RT 9.80 min.

## Conclusions

The MN exploratory analyses indicated chemical differences among the extracts from different brown and red algae species, and among different collection sites for the same species, including sites closer to the coast (LC1 and LC2) and on the oceanic islands. For some extracts it was possible to identify mass values compatible with metabolites already reported in the literature, such as terpenoids (F1, F3, F4 and F7 besides individual nodes) and C_15_-acetogenins (F7 and individual nodes) already isolated from those or related species. Furthermore, one diterpene and three sesquiterpenes were isolated from *Dictyota mertensii* (DM2) and *Laurencia catarinensis* (LC2) extracts, respectively. Due to the limitations of mass spectrometry for structure assignment, the metabolites reported here could also have an isomeric skeleton of the proposed structure. Many nodes in the MN could not be characterized, and these metabolites could be used as targets for isolation in a future work.

## Conflicts of interest

There are no conflicts to declare.

## Supplementary Material

RA-008-C8RA02802H-s001
