# Supplementary material for: Molecular networking prospection and characterization of terpenoids and C15-acetogenins in Brazilian seaweed extracts
Source: RSC Adv. 2018 Aug 21;8(52):29654–61. doi: 10.1039/c8ra02802h (PMC9085288; doi:10.1039/c8ra02802h)
Supplement: RA-008-C8RA02802H-s001 [file RA-008-C8RA02802H-s001.pdf]

Electronic Supplementary Material (ESI) for RSC Advances.
This journal is © The Royal Society of Chemistry 2018

Supporting Information

Molecular Networking prospection and characterization of terpenoids and C_{15}-acetogenins from the Brazilian seaweeds

Ana Cláudia Philippus, †[a] Gabriele A. Zatelli, †[a] Tauana Wanke, [a] Maria Gabriela A. de Barros,[a] Satomy A. Kami,[a] Cintia Lhullier,[a] Lorene Armstrong, [a] Louis P. Sandjo [a] and Miriam Falkenberg *[a]

[a] Federal University of Santa Catarina (UFSC), Postgraduate Program in Pharmacy, Health Sciences Center, Florianópolis, 88.040-900, Brazil. E-mail:miriam.falkenberg@gmail.com

Supporting Information List

Page S2-4. ^{1}H, ^{13}C, HSQC and HMBC NMR spectra of compound **9** (pachydictyol A).

Page S5-6. ^{1}H, ^{13}C NMR spectra, and IR experiment of compound **20** (prepacifenol epoxide).

Page S7-9. ^{1}H, ^{13}C, HSQC, HMBC and COSY NMR spectra, and IR experiment of compound **21** (johnstonol).

Page S10-12. ^{1}H, ^{13}C, HMBC and COSY NMR spectra, and IR experiment of compound **27** (pacifenol).

Page S13. Box S1. Compounds related to nodes.

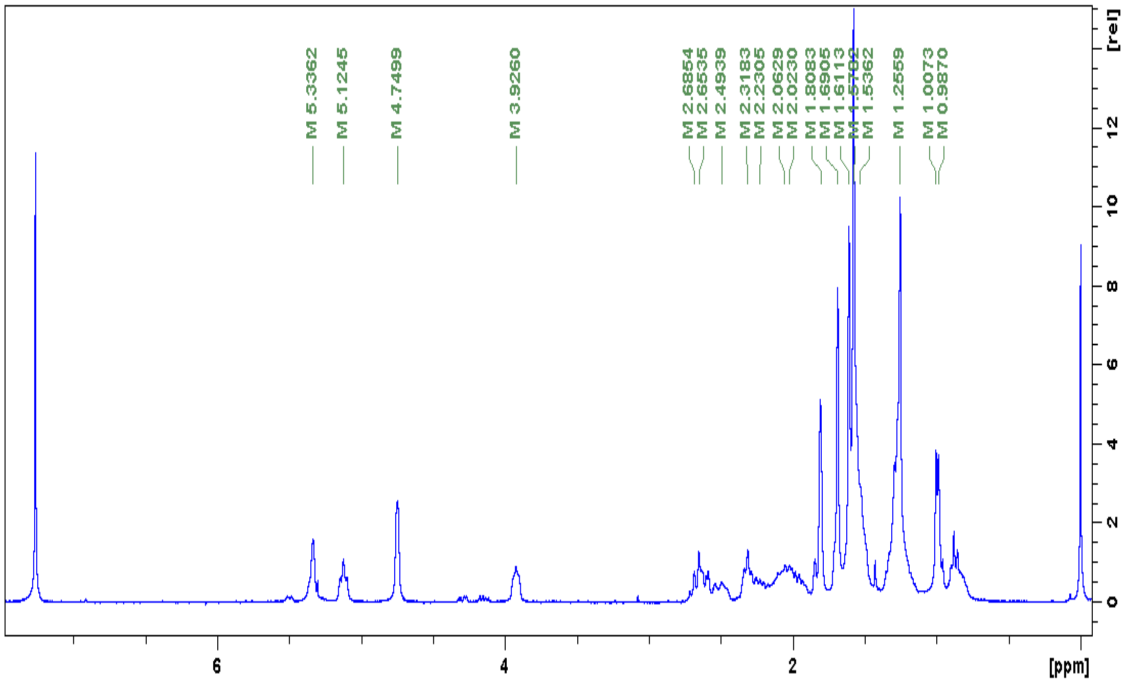

Figure **S1**. ^{1}H (400 Hz) NMR Spectrum of pachydictyol A (**9**) in $CDCl_3$.

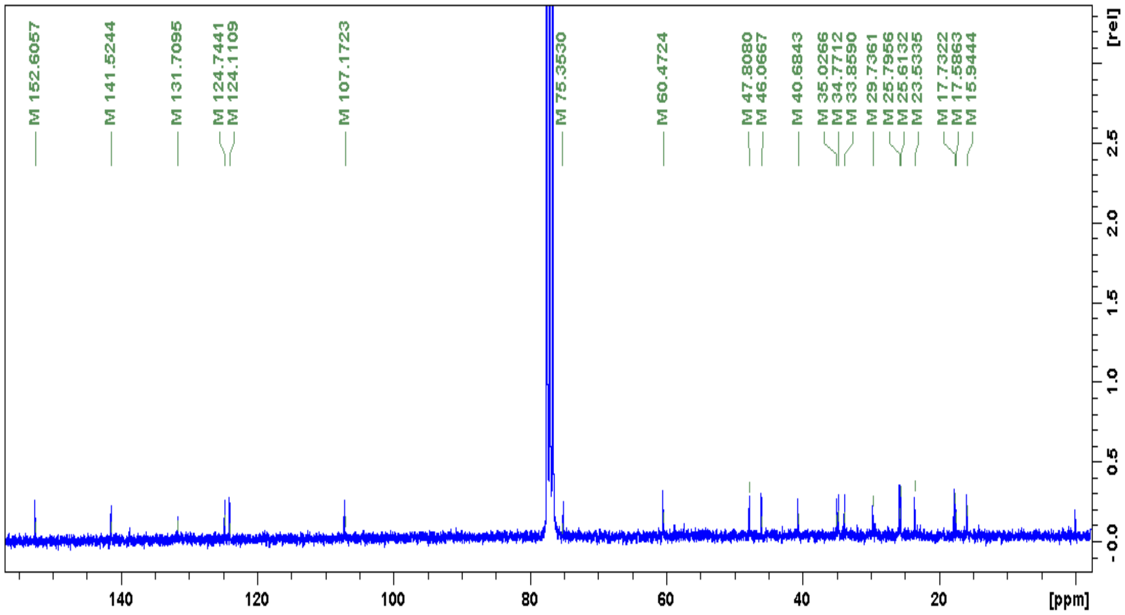

Figure **S2**.^{13}C (400 Hz) NMR Spectrum of pachydictyol A (**9**) in $CDCl_3$.

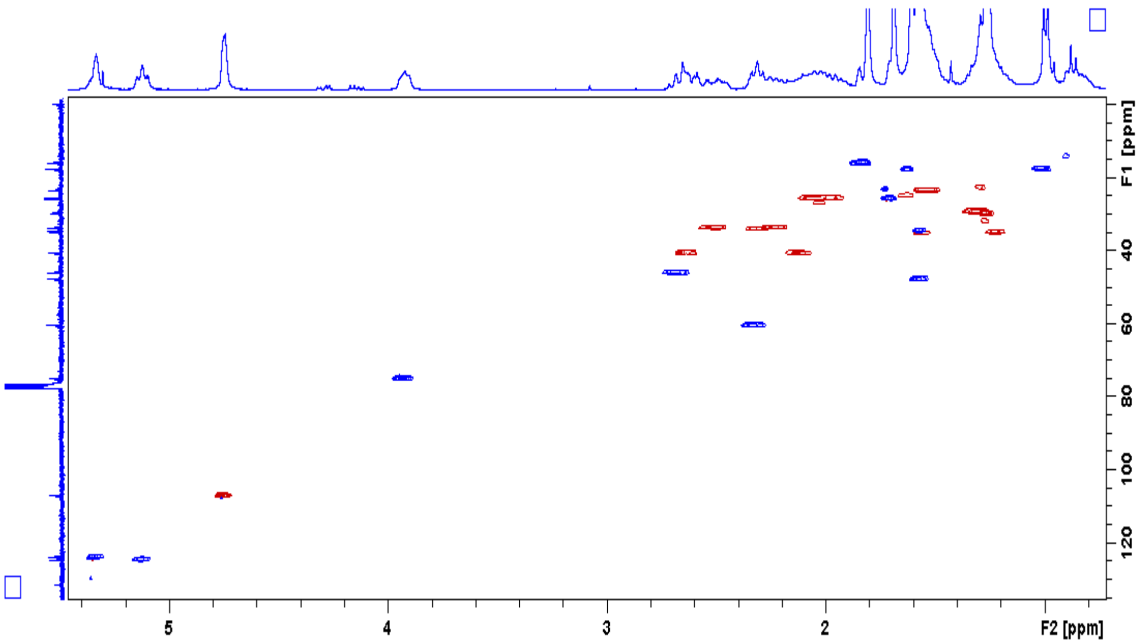

Figure **S3**. HSQC (400/100 Hz) NMR Spectrum of pachydictyol A (**9**) in $CDCl_3$.

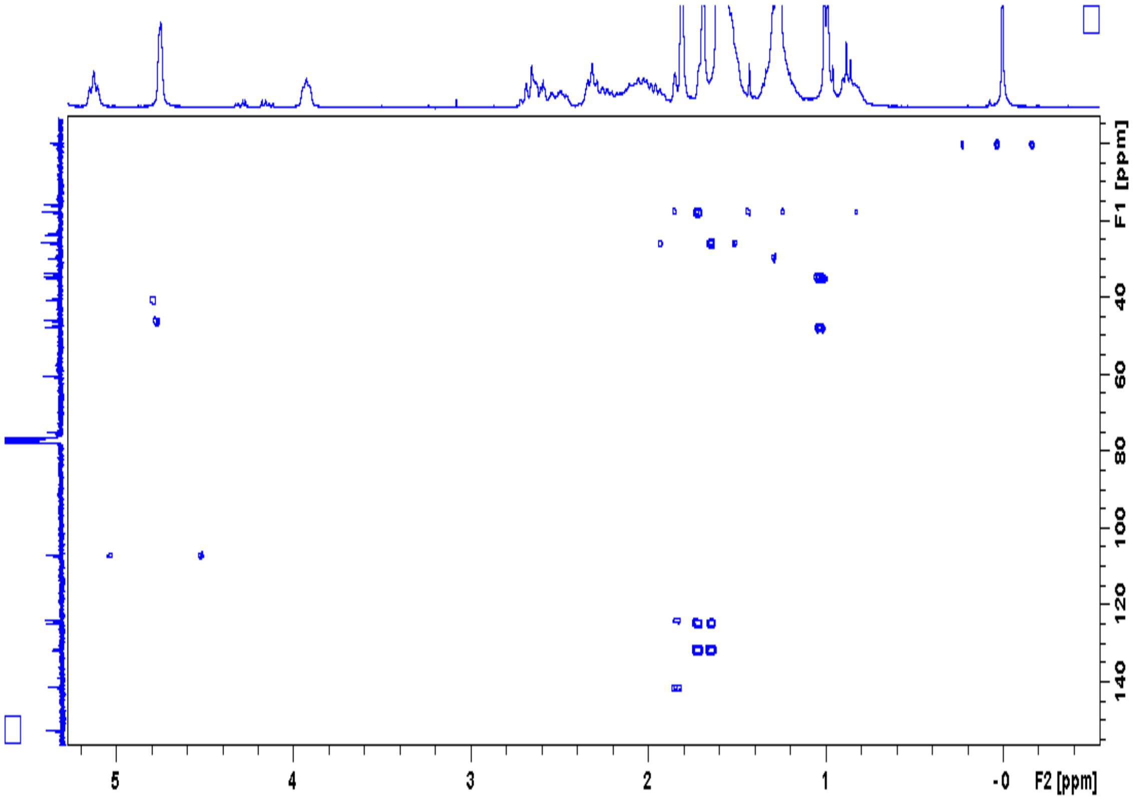

Figure **S4**. HMBC (400/100 Hz) NMR Spectrum of pachydictyol A (**9**) in $CDCl_3$.

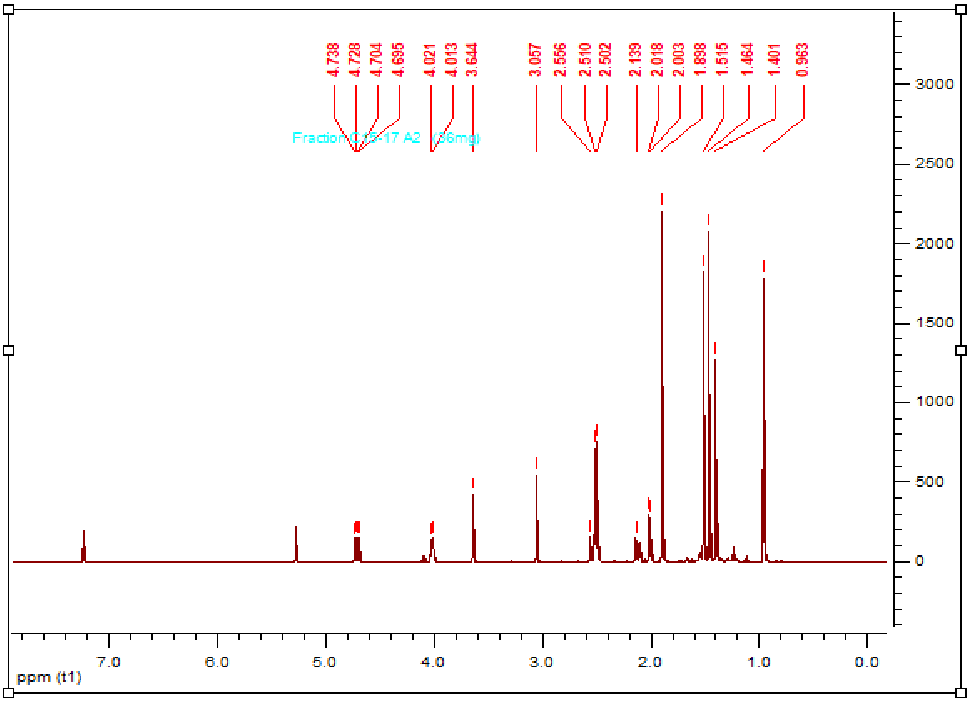

Figure **S5**. ^{1}H (400 Hz) NMR Spectrum of prepacifenol epoxide (**20**) in $CDCl_3$.

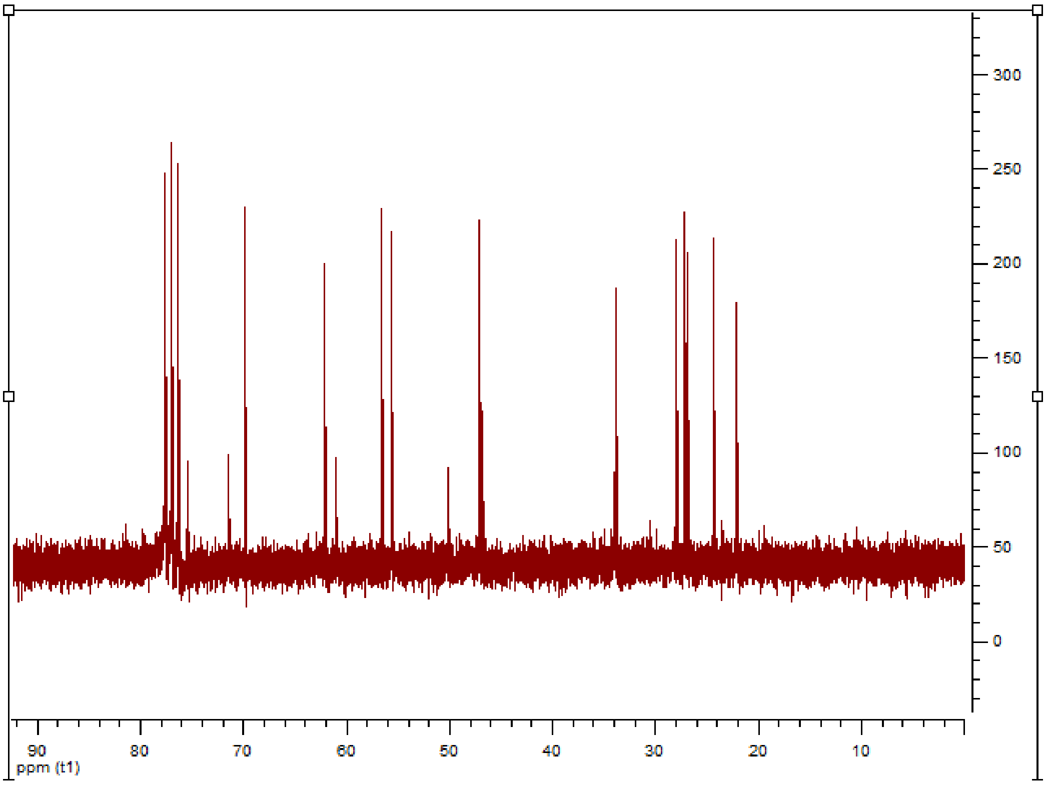

Figure **S6**.^{13}C (400 Hz) NMR Spectrum of prepacifenol epoxide (**20**) in $CDCl_3$.

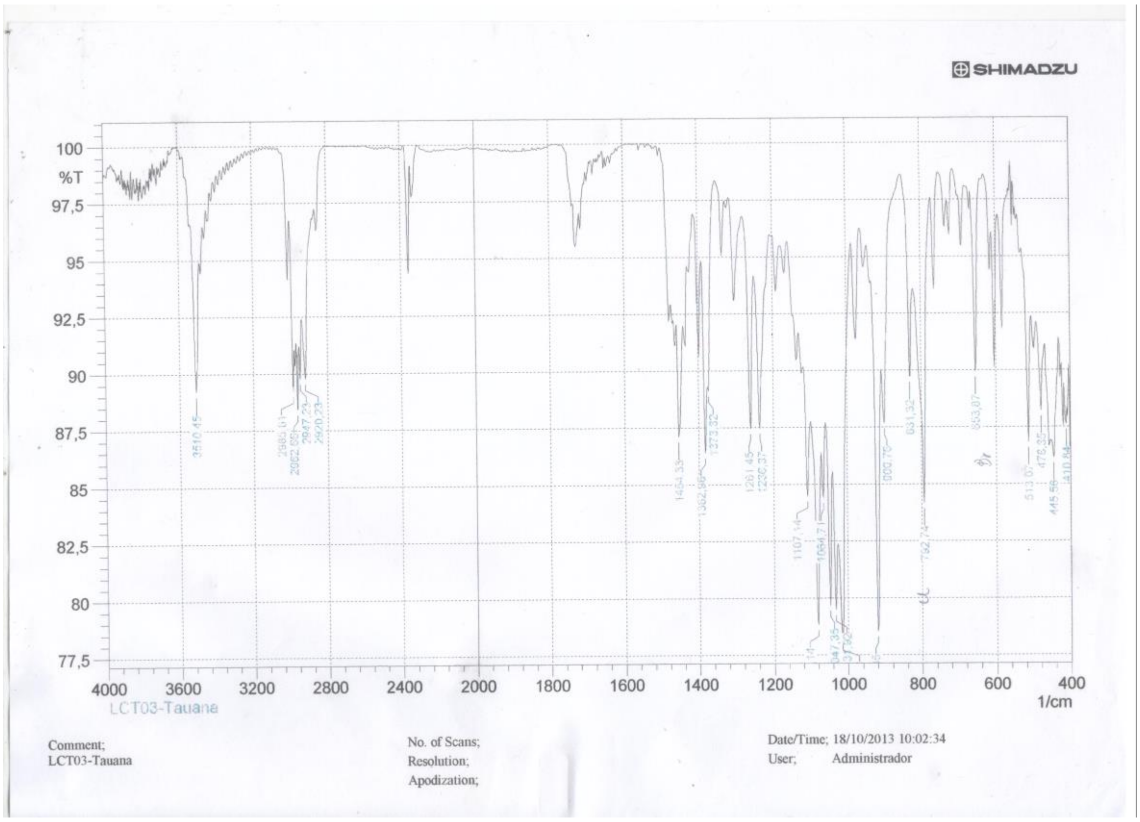

Figure **S7**. IR spectrum of prepacifenol epoxide (**20**)

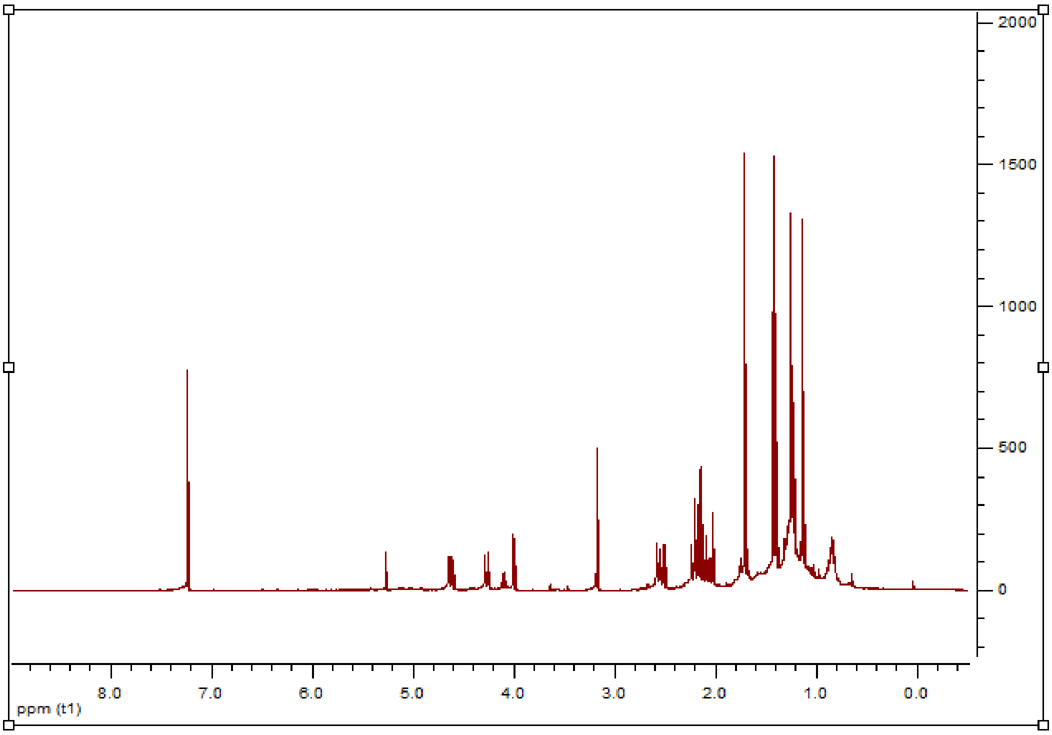

Figure **S8**. ^{1}H (400 Hz) NMR Spectrum of johnstonol (**21**) in $CDCl_3$.

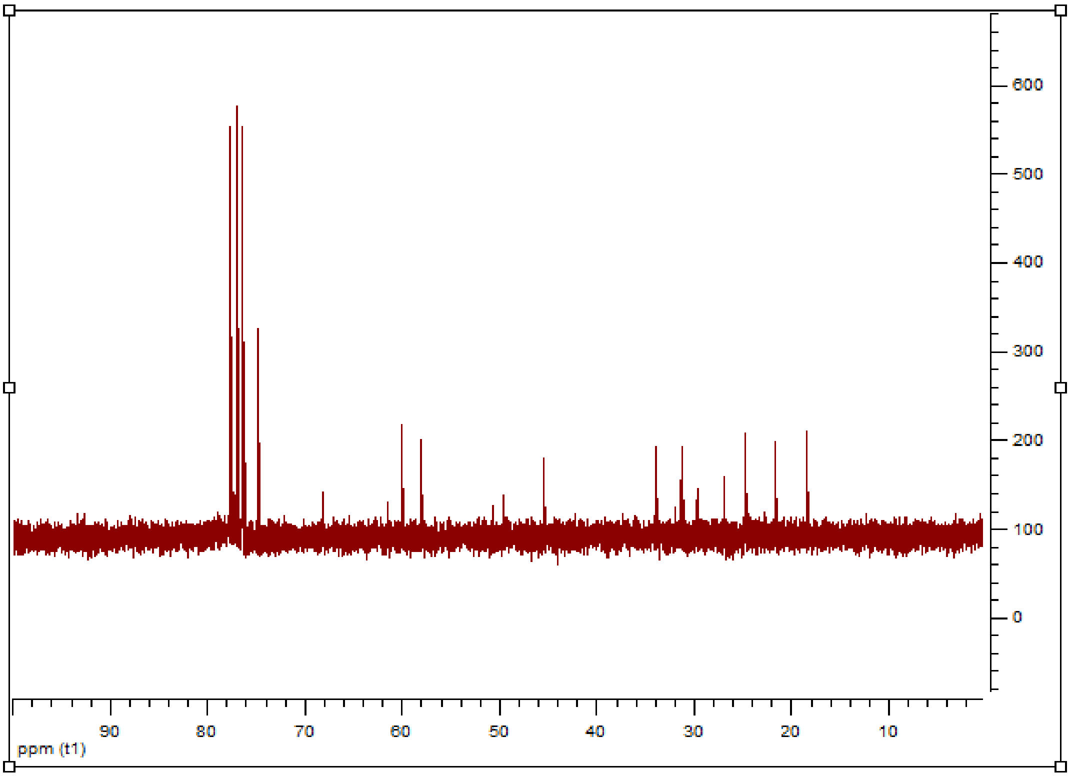

Figure **S9.**^{13}C (400 Hz) NMR Spectrum of johnstonol (**21**) in $CDCl_3$

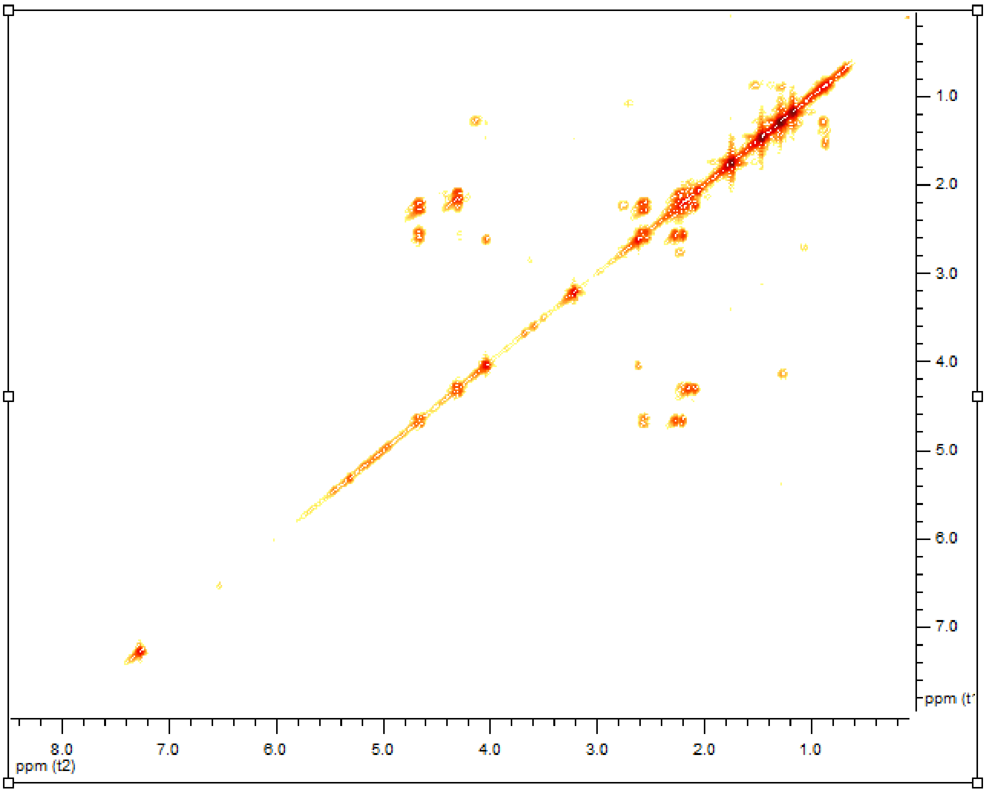

Figure **S10**. ^{1}H-^{1}H COSY (400 Hz) NMR Spectrum of johnstonol (**21**) in $CDCl_3$.

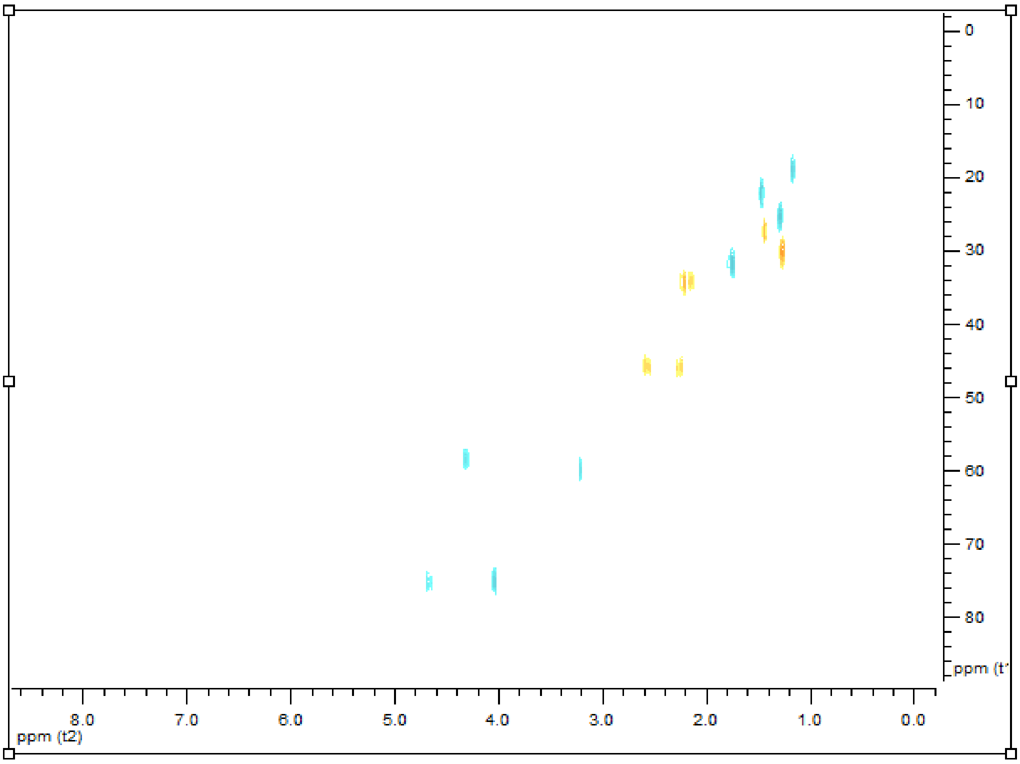

Figure **S11**. HSQC (400/100 Hz) NMR Spectrum of johnstonol (**21**) in $CDCl_3$.

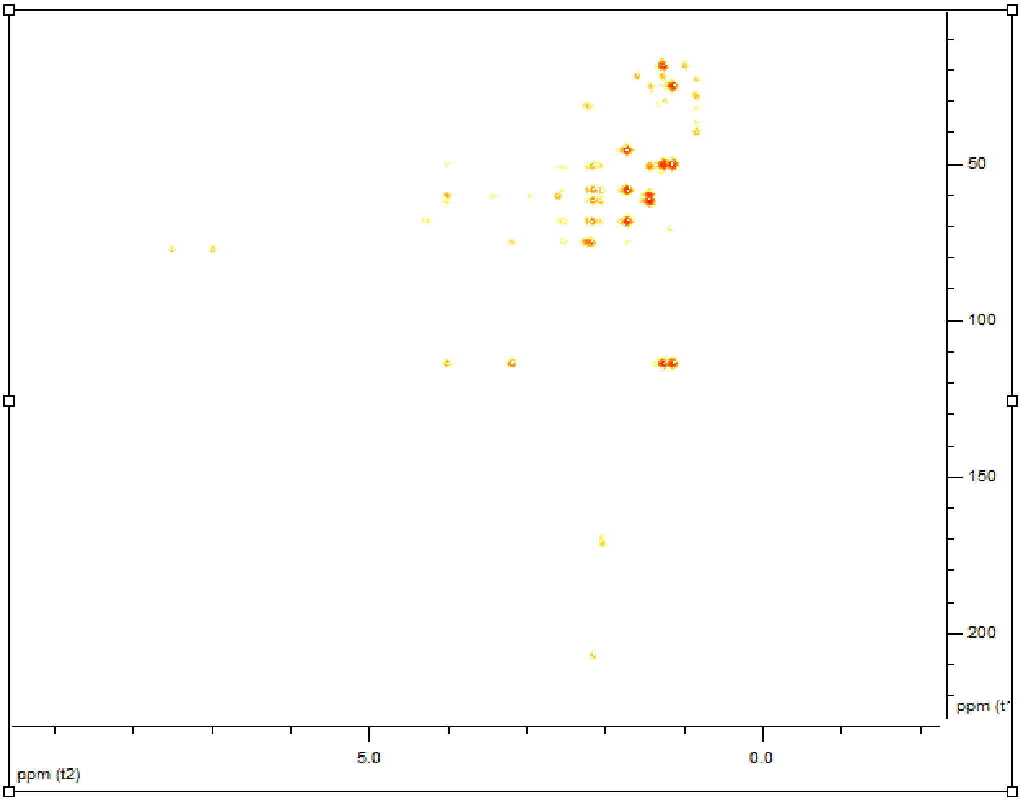

Figure **S12**. HMBC (400/100 Hz) NMR Spectrum of johnstonol (**21**) in $CDCl_3$.

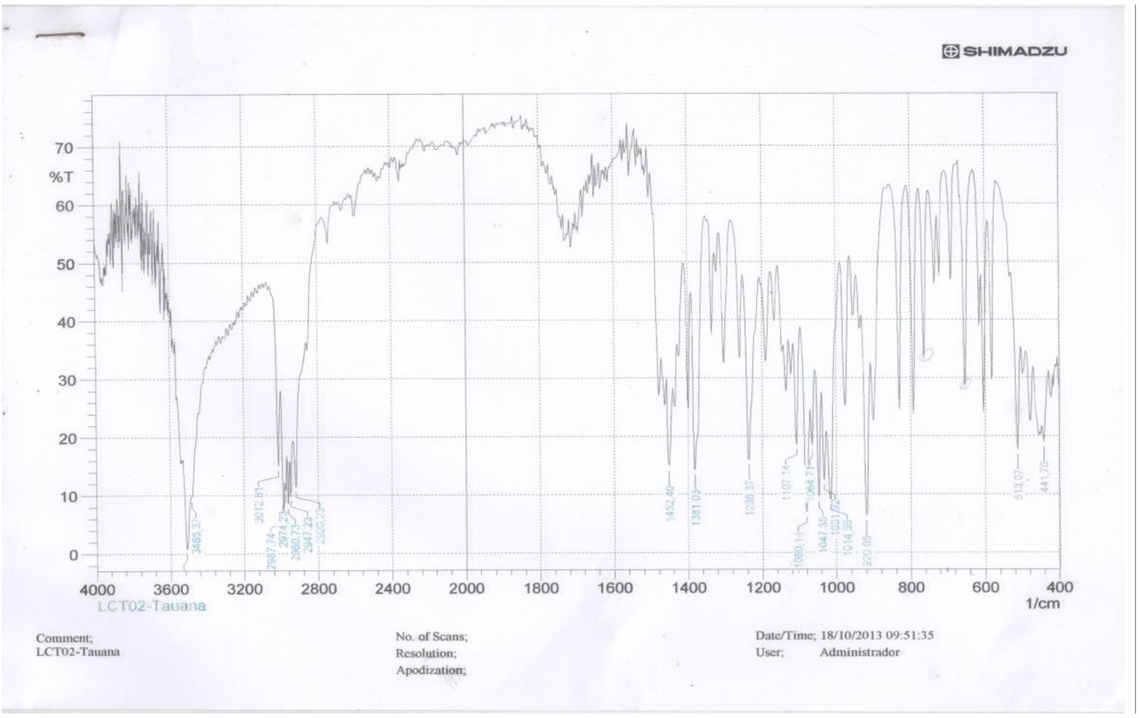

Figure **S13**. IR spectrum of johnstonol (**21**)

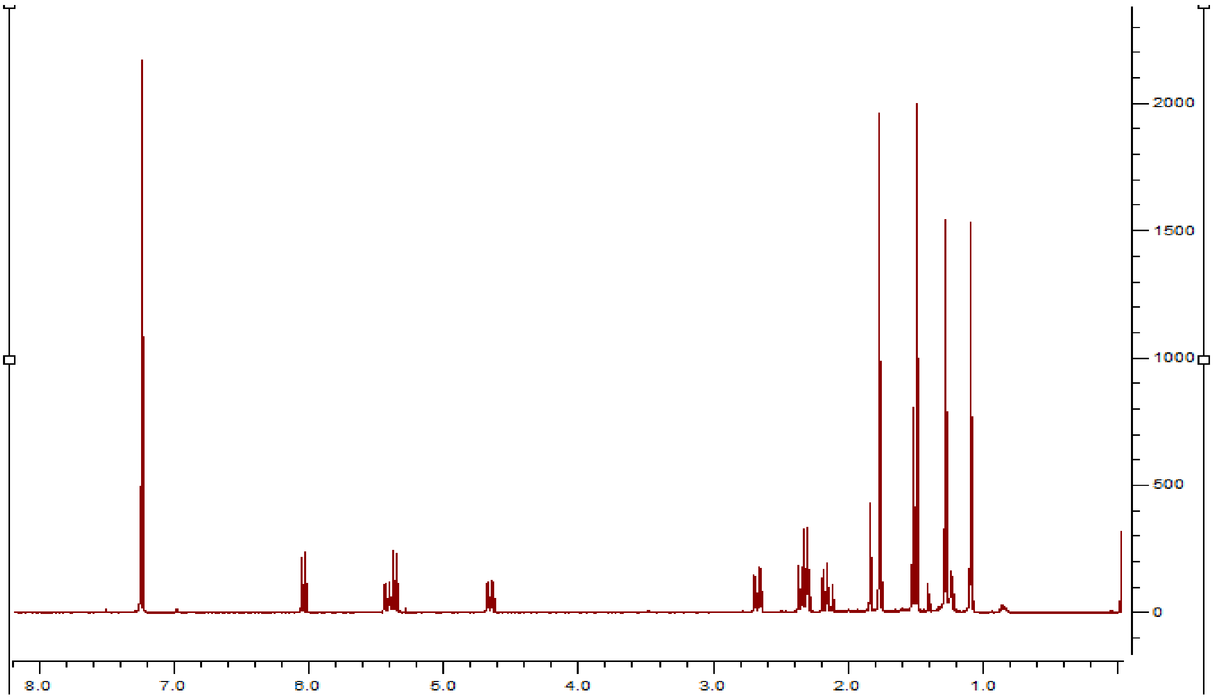

Figure **S14**. ^{1}H (400 Hz) NMR Spectrum of pacifenol (**27**) in $CDCl_3$.

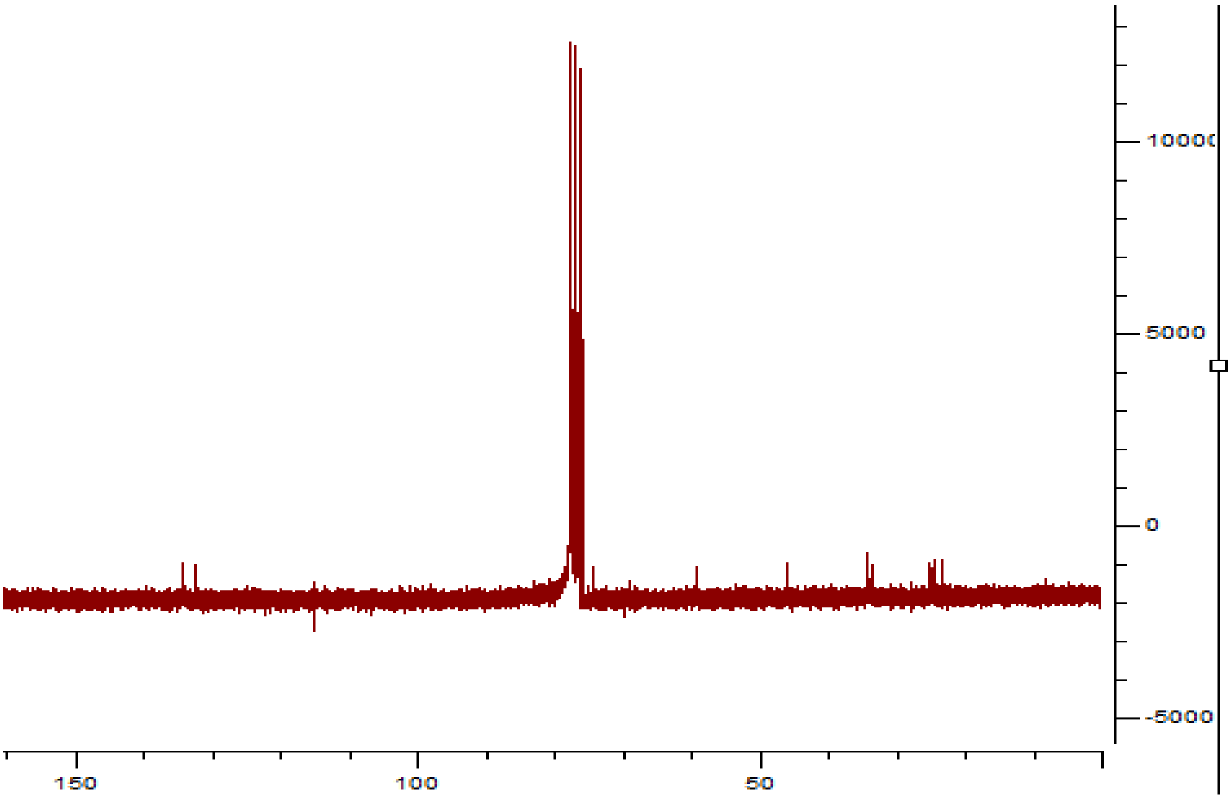

Figure **S15.**^{13}C (400 Hz) NMR Spectrum of pacifenol (**27**) in $CDCl_3$.

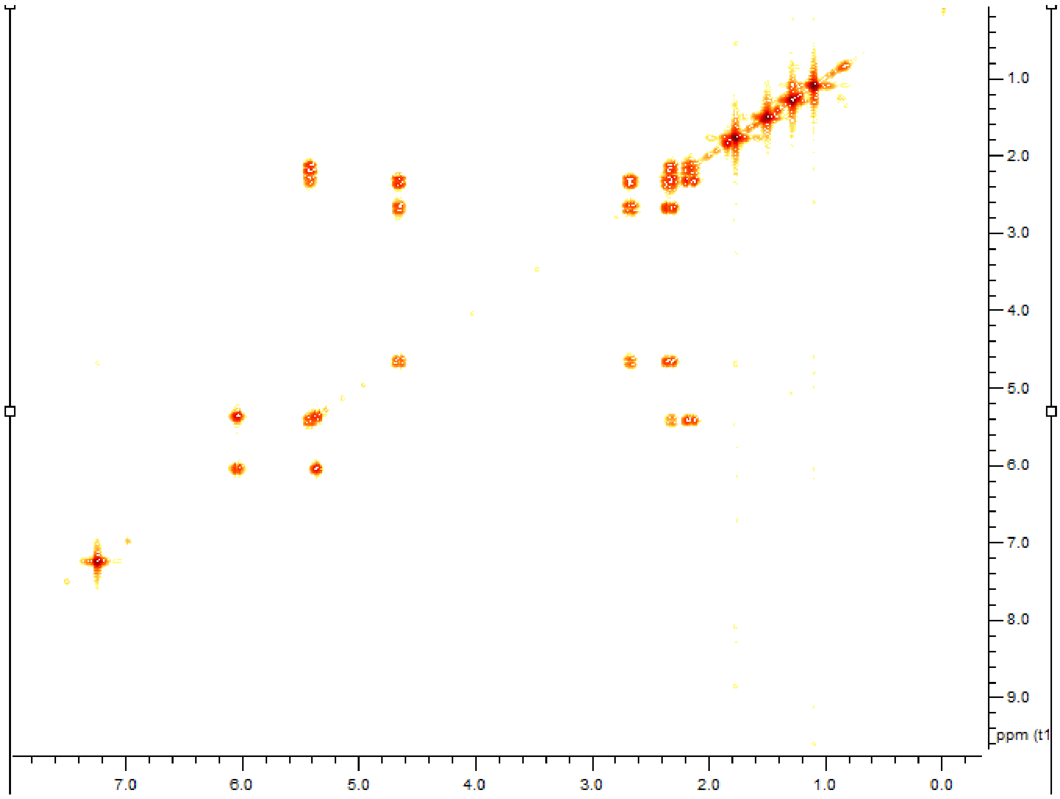

Figure **S16**. ^{1}H-^{1}H COSY (400 Hz) NMR Spectrum of pacifenol (**27**) in $CDCl_3$.

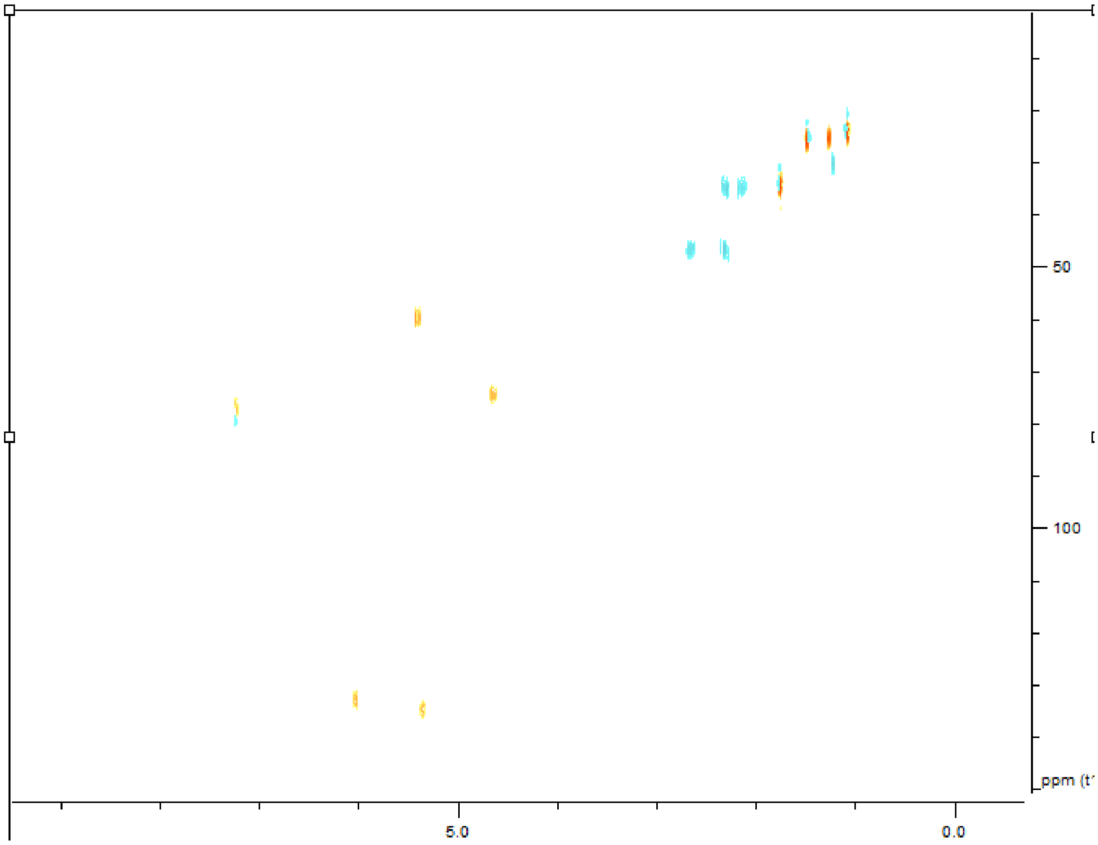

Figure **S17**. HSQC (400/100 Hz) NMR Spectrum of pacifenol (**27**) in $CDCl_3$.

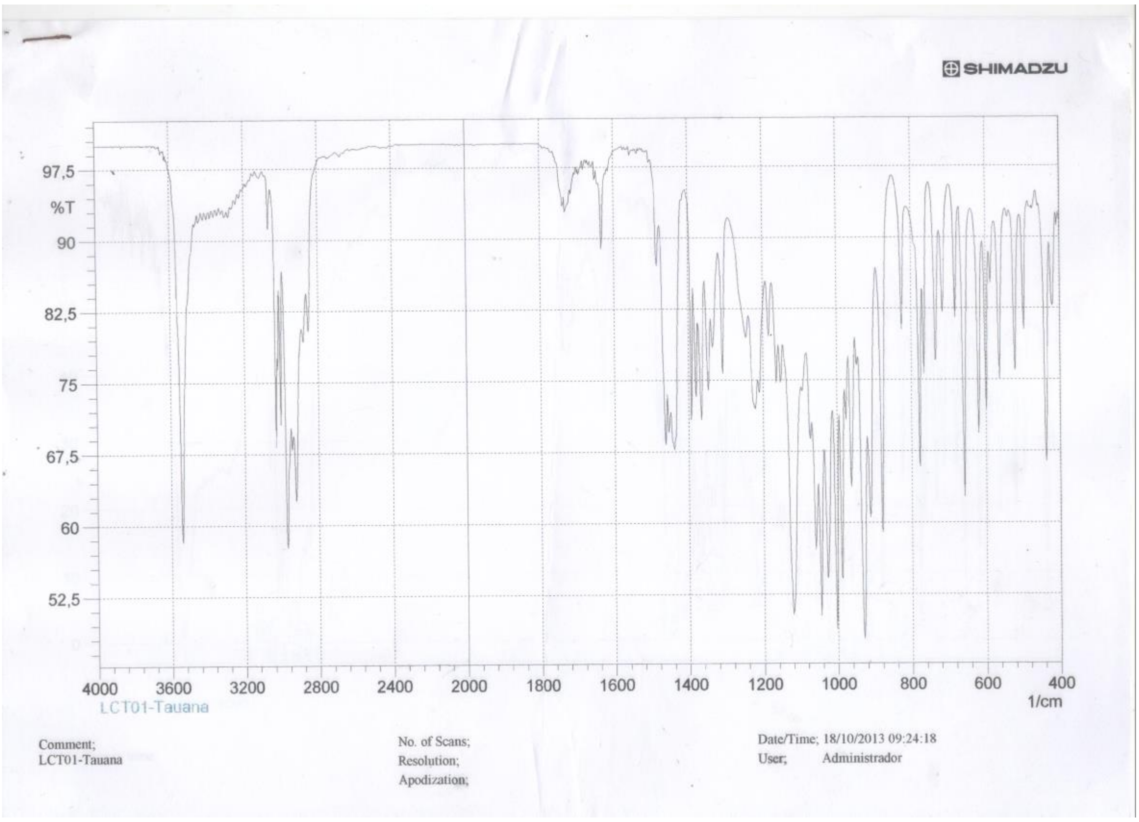

Figure **S18**. IR spectrum of pacifenol (**27**)

Box S1. Compounds related to nodes.

Family	Node	Extract Code	Compound	Class	Reference
**F1**	*m/z* 319.2274	CC1, CC2, DM1, DM3	4β-hydroxydictyodial (**1**)	Xeniane diterpene	14
	m/z 361.2383<br>*m/z* 301.2167	CC1, DM1	4β-acetoxydictyodial A (**2**)	Xeniane diterpene	13
	m/z 321.2441<br>*m/z* 303.2332	CC1, CC3, DM2, DM3	18,4-dihydroxydictyo-19-al A (**3**)	Xeniane diterpene	14
	m/z 267.2134	CC3, DM1	18-acetoxy-4-hydroxydictyo-19-al A (**4**)	Xeniane diterpene	14
	m/z 269.2265<br>*m/z* 270.2309	CC1, CC2, DM2	dictyotadiol (**5**)	Prenylated guaiane<br>diterpene	15
			dictyol B (**6**)	Prenylated guaiane diterpene	13
	m/z 369.2395	CC2, DM2	dictyol B acetate (**7**)		
	m/z 271.2439<br>*m/z* 272.2452	CC1, CC2, DM2	dictyoxide (**8**)	Prenylated guaiane diterpene	12
			pachydictyol A (**9**)	Prenylated guaiane diterpene	12
**F2**	-	CC2, CC3, DM2, DP1, DP3	-	-	-
**F3**	*m/z* 412.9883,<br>*m/z* 414.9859<br>*m/z* 416.9840	LC2	9-hydroxy-4,10-dibromo-3-chlorochamigrene (**10**)	Chamigrane sesquiterpene	21,22,23
			2,10-dibromo-3-chloro-8-hydroxychamigrene (**11**)	Chamigrane sesquiterpene	24
			laucapyranoid A (**12**)	Bisabolane sesquiterpene	25
**F4**	m/z 412.9717,<br>m/z 414.9692<br>m/z 432.9804	LC2	laucapyranoid B (**13**)	Bisabolane sesquiterpene	25
			laucapyranoid C (**14**)	Bisabolane sesquiterpene	25
			4,10-dibromo-3-chloro-7,8-epoxy-9-hydroxychamigrane (**15**)	Chamigrane sesquiterpene	25

			4,10-dibromo-3-chloro-7,8-epoxychamigrane (**16**)	Chamigrane sesquiterpene	26
			4,10-dibromo-3-chloro-7,8-epoxy-5-hydroxychamigrane (**17**)	Chamigrane sesquiterpene	27
**F5**	-	DM1, DM2, CC1, DP1	-	-	-
**F6**	-	DM1, DM3	-	-	-
**F7**	*m/z* 390.9736	LC1	laurendecumallene A (**18**)	C_{15}-acetogenin	29
			laurendecumallene B (**19**)	C_{15}-acetogenin	29
	m/z 408.9829	LC1	prepacifenol epoxide (**20**)	Chamigrane sesquiterpene	30
			johnstonol (**21**)	Chamigrane sesquiterpene	21
**F8**	-	CC2, DM2	-	-	-
**F9**	-	CC1	-	-	-
**Individual node**	*m/z* 392.9902	LC2	laureepoxide (**22**)	C_{15}-acetogenin	33
			(3*E*)-elatenyne (**23**)	C_{15}-acetogenin	29,34
			elatenyne (**24**)	C_{15}-acetogenin	29,34
			kumausallene (**25**)	C_{15}-acetogenin	24
			laurobtusin (**26**)	C_{15}-acetogenin	35
**Individual node**	*m/z* 408.9581 *m/z* 410.9542	LC2	pacifenol (**27**)	Chamigrane sesquiterpene	37,38,39
